# Plantar erythrodysesthesia with bullous otitis externa, toxicities from sorafenib: a case report

**DOI:** 10.4076/1757-1626-2-6264

**Published:** 2009-09-16

**Authors:** Corey A Carter, Arun Rajan, Giuseppe Giaccone

**Affiliations:** 1 CCR, National Cancer Institute10 Center Drive, Building 10, Room 12N226, Bethesda, MD 20892USA; 2 Medical Oncology Branch, CCR, National Cancer Institute10 Center Drive, Building 10, Room 12N226, Bethesda, MD 20892USA

## Abstract

Lung cancer is the leading cause of cancer death worldwide and the use of novel agents, such as sorafenib has now demonstrated activity in Non Small Cell Lung Cancer. We present a case of a 77-year-old Caucasian male with advanced adenocarcinoma of the lung, who was being treated on clinical trial with single agent sorafenib. After seven weeks of treatment the patient presented to clinic with difficulty walking. Physical exam revealed acral erythema with bollous formation on bilateral soles of his feet. Otoscopic exam revealed bilateral external canal bullous lesion. The patient was diagnosed with plantar erythrodysesthesia with bullous otitis externa, a new toxicities in patients being treated with sorafenib.

## Introduction

Lung cancer is the leading cause of cancer death worldwide [1]. Use of novel agents, such as sorafenib, a multitargeted receptor tyrosine kinase inhibitor, have demonstrated activity in non small cell lung cancer (NSCLC) [2-3]. Currently, the toxicities reported in NSCLC have been similar to those reported in Renal Cell Carcinoma clinical trials [4]. Here we present a case of new toxicity that occurred on a relatively low dose of sorafenib.

## Case presentation

**Figure 1.**
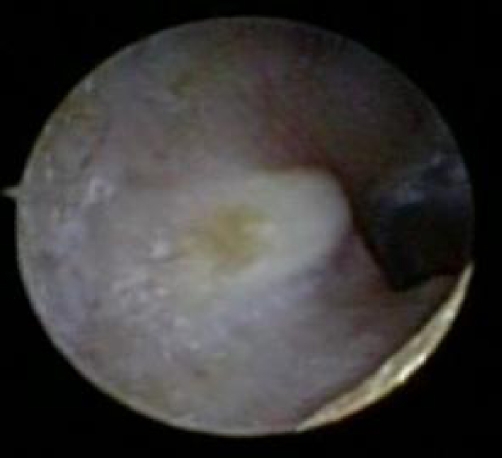
Otoscopic exam of the right external auditory canal. A small 0.4 cm bullous lesion, with no surrounding erythema.

**Figure 2.**
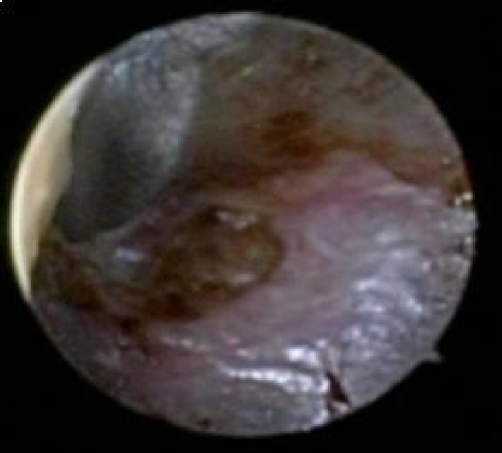
Otoscopic exam of the left external auditory canal. A 1.0 cm ruptured bullous lesion with associated bleeding of the bullous.

A 77-year-old Caucasian male with advanced adenocarcinoma of the lung, was being treated on clinical trial with single agent sorafenib. He was started on 400 mg orally twice a day and developed grade 3 hypertension after two weeks of therapy and was subsequently dose reduced to 200 mg orally twice a day. During his fifth week of treatment the patient reported to a mild decrease in his hearing as well as mild facial erythema, grade 1. Otoscopic examination revealed bilateral mild erythematous skin in the external auditory canal without evidence of skin breakdown and bilateral impacted cerumen which was managed with warm saline washes resulting in resolution of his hearing deficit. After seven weeks of treatment the patient presented to clinic with an acute complaint of difficulty walking. Physical exam revealed acral erythema with bollous formation along the pressure points of bilateral soles of his feet. Otoscopic exam of bilateral external auditory canal revealed a right ulcerated lesion (Figure 1) and the left ear showed a ruptured bullous lesion (Figure 2). Biopsy of the left ear lesion revealed no evidence of necrosis, as well as no evidence of a viral, bacterial, or fungal infection. Based on the clinical findings and without any history of ear canal trauma, a diagnosis of plantar erythrodysesthesia with bullous otitis externa associated with sorafenib was made. Sorafenib was withdrawn and all toxicities resolved to grade 1 within one week. Patient underwent scheduled imaging 8 weeks from the start of sorafenib, prior to reinstitution of the sorafenib, and was found to have progression of disease. He was subsequently discontinued from this clinical trial.

## Discussion

Sorafenib is a multi-tyrosine kinase inhibitor that targets serine/threonine and receptor tyrosine kinases and has been shown to decrease both tumor growth and angiogenesis [4]. Sorafenib has activity against the vascular endothelial growth factor receptor (VEGFR-2, VEGFR-3), platelet-derived growth factor receptor (PDGFR-beta), stem cell factor receptor (c-KIT), and the RET, BRAF and FLT-3 kinase pathways. The most common reported adverse events are diarrhea, rash, fatigue, hypertension and hand-foot-skin reactions [5].

Palmar and plantar erythrodysesthesia (hand-foot-skin reactions) has been reported to occur in up to 30% of patients being treated with monotherapy sorafenib for clear cell renal cell carcinoma [5]. The mechanism of which this syndrome occurs is unique from other acral chemotherapy reactions (hand-foot syndrome). Chemotherapies (i.e. liposomal doxorubicin, capecitabine) causing acral reactions are believed to be secondary to high concentrations of the drugs being secreted in the eccrine glands [6]. The human body has a high concentration of eccrine glands located in the palms and soles of the feet [6]. The acral toxicities secondary to multi-tyrosine kinase inhibitors occur in the palms and soles of the hands and feet but distinctly appear along the pressure points of the feet as well as along the sides and web areas of these surfaces. The severity of the lesions has been shown to be dose dependent and resolution of symptoms occurs with withdrawal of the drug [7]. It has been proposed that this occurs in this distinct pattern, which is different from chemotherapy induced acral reactions, secondary to the combination of the vascular anatomy within these areas, the temperature gradient of these points and the increased concentration of the eccrine glands [8]. The feet and hands have a rich capillary network and use of the extremities cause increases in blood flow to these areas [8].

The cercum glands in the external auditory canal are a specialized variant of eccrine glands which function both as apocrine and eccrine glands [9]. The face is the most vascular dermal area of the body. The vascularity of the external auditory canal has been documented as having the largest skin temperature gradient [10]. This lends the external auditory canal to be at significant risk of developing skin toxicities associated with sorafenib. The development of bullous lesions in the external auditory canal is supportive of previous proposed mechanisms of dermal skin toxicities associated with sorafenib with the exception of being a pressure point. Our patient developed blockage of the ear canal secondary to cercum build up which led to irrigation and increased manipulation of the external auditory canal. His acute bullous otitis externa was asymptomatic and responded to withdrawal of the offending agent.

## Conclusion

The list of toxicities of multitargeted tyrosine kinase inhibitors is growing as use increases in more solid tumors. Skin toxicities are common when using multitargeted tyrosine kinase inhibitors with up to one-third of patients developing grade 3 toxicities. Otoscopic examination should be considered in patients that develop hand-foot and skin toxicities and complain of hearing disturbances.

## Abbreviations

BRAF: -V-raf murine sarcoma viral oncogene homolog B1
c-KIT: stem cell factor receptor
FLT-3: Fms-related tyrosine kinase 3 ligand three
NSCLC: non small cell lung cancer
PDGFR: platelet derived growth factor receptor
RET: rearranged during transfection
VEGFR-2: vascular endothelial growth factor receptor two
VEGFR-3: vascular endothelial growth factor receptor three
